# Edge-to-Cloud IIoT for Condition Monitoring in Manufacturing Systems with Ubiquitous Smart Sensors

**DOI:** 10.3390/s22155901

**Published:** 2022-08-07

**Authors:** Zhi Li, Fei Fei, Guanglie Zhang

**Affiliations:** 1 College of Computer Science and Software Engineering, Shenzhen University, Shenzhen 518060, China; 2 College of Automation Engineering, Nanjing University of Aeronautics and Astronautics, Nanjing 211100, China; 3 City University of Hong Kong Shenzhen Research Institute, Shenzhen 518057, China

**Keywords:** Industrial IoT, condition monitoring, anomaly detection, unsupervised learning approach, relative wavelet energy, LSTM

## Abstract

The Industrial Internet of Things (IIoT) connects industrial assets to ubiquitous smart sensors and actuators to enhance manufacturing and industrial processes. Data-driven condition monitoring is an essential technology for intelligent manufacturing systems to identify anomalies from malfunctioning equipment, prevent unplanned downtime, and reduce the operation costs by predictive maintenance without interrupting normal machine operations. However, data-driven condition monitoring requires massive data collected from smart sensors to be transmitted to the cloud for further processing, thereby contributing to network congestion and affecting the network performance. Furthermore, unbalanced training data with very few labelled anomalies limit supervised learning models because of the lack of sufficient fault data for the training process in anomaly detection algorithms. To address these issues, we proposed an IIoT-based condition monitoring system with an edge-to-cloud architecture and computed the relative wavelet energy as feature vectors on the edge layer to reduce the network traffic overhead. We also proposed an unsupervised deep long short-term memory (LSTM) network module for anomaly detection. We implemented the proposed IIoT condition monitoring system for a manufacturing machine in a real shop site to evaluate our proposed solution. Our experimental results verify the effectiveness of our approach which can not only reduce the network traffic overhead for the IIoT but also detect anomalies accurately.

## 1. Introduction

With the advances in the smart sensor and Industrial Internet of Things (IIoT) technologies, the development of intelligent manufacturing has been accelerated dramatically [1,2,3]. In modern manufacturing, intelligentization can help manufacturers at present to rise to the challenge of remaining ahead in the highly competitive market. The continuously improved sensor technology and data processing capability with big data, artificial intelligence, cloud computing, and edge technology are widely used in industrial equipment, process control, and factory management, leading to the further development of intelligent manufacturing. Intelligent manufacturing combines real-time data analysis, artificial intelligence (AI), and other machine learning technologies in manufacturing to improve production quality, reliability, and resource efficiency [4,5,6]. Sensors are attached to the manufacturing machines to collect and process real-time data through sensor networks for intelligent manufacturing, whereas AI algorithms based on the acquired data are designed to coordinate all operations of the manufacturing, such as equipment maintenance, manufacturing practices, and final product testing.

The condition of manufacturing systems needs to be monitored because they have become more complex with multiple functions [7,8]. A malfunctioning component can halt the manufacturing system. Information acquisition and analysis techniques are required to face these challenges because manufacturing systems have become more complex. Condition monitoring (CM) observes machinery condition parameters to identify changes and anomalies that indicate developing faults, reduce unexpected machine downtime, and ensure effective maintenance through intelligent manufacturing technologies [9,10], thereby reducing manufacturing costs and improving product quality to maintain competitiveness.

The IIoT extends information technology to operational technology by combining networked sensors and intelligent devices for applications in manufacturing systems, including data collection to drive artificial intelligence and adding intelligence to manufacturing equipment, processes, and management [11]. IIoT technology uses sensor nodes to collect massive data, improve production and manufacturing efficiency with the minimal cost through manufacturing operation visibility and data analysis, and continuously optimize manufacturing processes. The implementation of the IIoT is expected to help industrial manufacturing enterprises improve productivity, grow businesses, and improve customer service. IIoT sensors help the platform to collect data from the various modules of the system and allow our packaging equipment to be more efficient and productive. IIoT sensor nodes monitor the manufacturing process, evaluate the status of environmental conditions, predict the potential safety threats of systems, and allow critical maintenance operations to be completed online. These data can then be transferred to the controller of the system and processed by powerful analytics software that enables manufacturing modules to optimize manufacturing operations for improved performance. The IIoT platform is used to perform predictive analytics for the maintenance of the manufacturing system. Predictive analytics can identify failures and estimate the operating life of specific components with IIoT sensors, continuously collecting the sensor data of the system to monitor its status. Based on this data analysis, manufacturing machines can be repaired, or their components can be replaced in advance to minimize disruptions.

To obtain a more stable and efficient IIoT system, edge computing devices are deployed between cloud servers and the IIoT sensors and close to the sensors on the manufacturing machines to monitor the machine’s condition [12,13]. Edge devices can collect, process, and store data in a more distributed fashion and closer to the endpoints, hastening response times, reducing latency, and conserving network resources. In addition, edge computing reduces data transmission and network traffic. The distributed edge computing devices cause low latency processing to the shop floor and offload computing from the cloud servers to the edge devices. However, the computational capabilities and resources of edge devices are limited. Thus, an on-device program of the devices can help data preprocessing to reduce the data transmission and algorithm computing on the cloud servers efficiently.

Anomaly detection can identify the deteriorating condition of the machine by observing the exceptional patterns during the normal operation of the equipment. The anomalies are the early signs of system failure, which may lead to systematic faults or equipment breakdown [14,15]. However, only 20 percent of all anomalies in manufacturing are predicted and understood beforehand [16]. Vibration analysis is a fundamental method to monitor and diagnose the machine’s condition with the vibration amplitude and frequency, given that the vibration sensor can be installed on the machines to monitor system conditions without breaking the structures. Static thresholding is one of the simplest techniques to identify anomalous sequences within a time series; an alert is raised whenever a data point exceeds the expected range. However, this approach often fails to detect contextual anomalies because the machine’s condition changes.

Traditional statistical methods are used for anomaly detection at the early stages. Statistical anomaly detection techniques assume that normal data instances occur in high probability regions of a stochastic model, whereas anomalies occur in the low probability regions of the stochastic model [17]. However, selecting the best statistic to construct hypothesis tests for complex distributions with high dimensional data sets is very difficult. The traditional methods of anomaly detection cannot deal well with the modern manufacturing industry problems.

Machine learning methods process data for anomaly detection with analytical model building automatically and simplify sensor dataset handling to detect the new types of anomalies by self-training. The machine learning methods for anomaly detection are classified into supervised and unsupervised approaches. Supervised algorithms are trained models with datasets that are already labeled as normal and abnormal. The algorithm then processes the real sensor data and detects anomalies based on the trained model. A supervised algorithm only detects the normal or abnormal categories on which it has been trained with the pre-labeled datasets. Thus, a supervised machine learning algorithm cannot recognize a pattern that has not been previously trained. The new type of anomaly should be trained by the supervised models continuously to obtain a more robust detection system for every possible data distribution and pattern. This condition is not always practical for real industrial manufacturing situations. Unsupervised machine learning algorithms, different from supervised learning approaches, learn the normal behavior without the pre-labeled datasets, and then statistical techniques are applied to determine if a specific data point is an anomaly because very few anomalies are available during the normal operations of the manufacturing. The unsupervised anomaly detection methods can detect any type of anomaly, even those never seen before.

The anomaly detection should work with large amounts of different data types, which are often unstructured. Therefore, traditional machine learning models face the challenges of dealing with these unstructured data. These models are trained on structured data that have input features with corresponding output labels, so the unstructured data cannot be directly used as input features. However, traditional machine learning methods build a single model of prediction for all anomaly detection entries, and the model parameters are difficult to fit through the high-dimensional data and sequence datasets. The datasets used by traditional methods to train the model may not contain abnormal signals because anomalies are rare in real manufacturing scenarios. In addition, anomalies that have occurred in the past may not be a perfect indication of problems occurring in the future because anomaly detection detects faults under time-varying operational conditions the in long-term running. Recently, many studies have been conducted with deep learning on condition monitoring for manufacturing machines, the fault features of which are automatically extracted from the sensor data by the deep learning methods, and the feature extractor is not reconstructed for every specific fault diagnosis. The deep learning approach uses the available data to learn the underlying model of the time series, enabling it to perform more complex anomaly detection tasks. However, most methods take directly raw time-series vibration signals as input. The large amount of data is a great burden on the system. The high volume of the sensor data is also challenging to the system’s ability for data transmission and processing. We proposed an edge-to-cloud condition monitoring platform for anomaly detection. The relative wavelet energy-based LSTM model is implemented, and the relative wavelet energy is computed on the edge layer to reduce the network traffic overhead and accelerate the model deployment.

The remainder of the paper is organized as follows: The methodology of the IIoT-based anomaly detection using relative wavelet energy and the LSTM model is discussed in Section 3. Section 4 describes the setup of the IIoT-based condition monitoring system deployed in a real shop site to validate the proposed anomaly detection method. The performance of the method is also discussed, based on the experimental results in this section. Finally, we conclude the study in Section 6.

## 2. Setup for Hardware Platform

**Figure 1:**
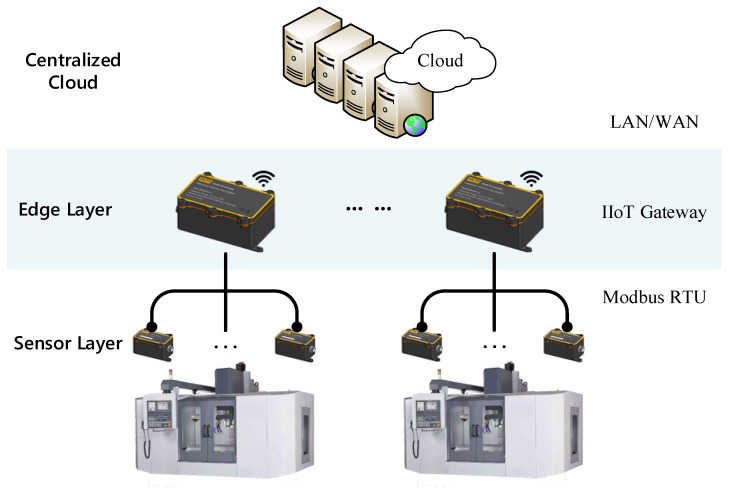
Overall architecture of an edge-to-cloud based IIoT condition monitoring system.

Machine condition monitoring is performed through IIoT devices organized as a hierarchy network. The proposed edge-to-cloud condition monitoring system with an unsupervised learning approach is illustrated in Figure 1. This system consists of three layers, including a centralized cloud, edge layer, and sensor layer. Smart sensor devices on the sensor layer collect the vibration signal on the manufacturing machines. The IIoT gateways of the edge layer implement the functions of signal conditioning, data acquisition, and signal preprocessing, whereas the unsupervised learning algorithms on the centralized cloud servers monitor the condition of the manufacturing machines and detect faults.

The smart sensor devices construct the sensor layer. Each sensor device consists of sensors, a signal conditioning circuit, communication components, a power management unit, and a microcontroller. The IIoT devices have been deployed in different places of the machines to collect sensor data in real-time. These sensor data represent the running state of the machines, and the devices transfer the sensor data through the IIoT sensor networks.

The IIoT gateway is deployed to connect the IIoT sensor network to the centralized cloud. To connect the cloud servers, the gateway supports a wired Ethernet connection and wireless networking protocols over the Internet. In addition, the gateway connects the smart sensor devices through RS485 using a Modbus RTU protocol. Modbus is a popular industrial protocol because it is simple and universal, especially for the compatibility of legacy industrial systems. This finding indicates that a Modbus connection can provide digital communications in older systems with new devices.

## 3. Methodology

**Figure 2:**
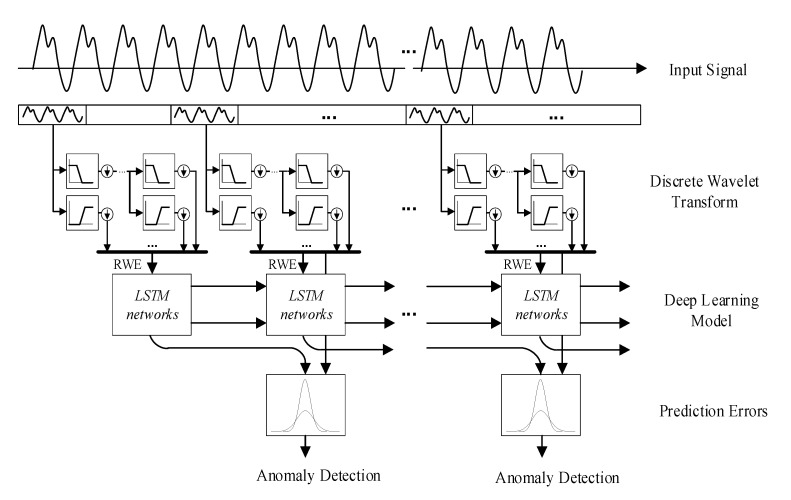
RWE–LSTM model-based anomaly detection algorithm.

The overview of the proposed anomaly detection algorithm is shown in Figure 2; it consists of a discrete wavelet transform and relative wavelet energy calculation module to extract local features of a short discrete sampling window and an LSTM module for estimating the long-term trends. In this section, we initially introduce how the input data are processed on the edge layer and how the LSTM module is trained with the relative wavelet energy as the features, and then explain how to implement anomaly detection finally.

### 3.1. Data Processing on the Edge Layer

Satisfying the IIoT data processing requirements by the traditional heterogeneous network architecture is very challenging because the massive sensor data are generated with the growth of the sensors’ number. Edge computing technology helps to extend cloud-computing capabilities to the edge layer of the network. It can manage the data transmission and enable data preprocessing to avoid network congestion and reduce the computing loads on the clouds.

The developing fault trend of the manufacturing systems is slow, whereas a high sampling frequency is required to capture the failure-generated signal, which is modulated at a high frequency. To maintain a trade-off between the sensitivity of failure detection and the number of data, we identify the optimal discrete sampling window size and interval to measure the sensor signal.

**Figure 3:**
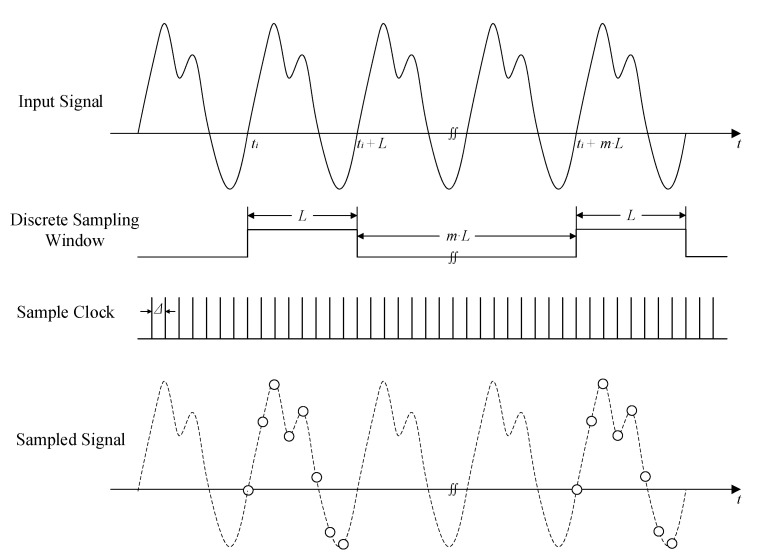
Signal digitization with discrete sampling window method.

The time series $X={\left\{x_{n}\right\}}_{n=1,2,\ldots N}$, which is a temporal sequence of the observations, is considered, and $x_{n}$ is a reading at the n-th time point of the sensor output. The output signal of the sensors in the time series reflects the working condition of the system. L is a discrete sampling window length. The sequence in the sampling window at time t_i_ is represented as $S\left(t_{i}\right)=\left[x_{t_{i}},x_{t_{i}+1},\cdots ,{\;x}_{t_{i}+\left(L-1\right)}\right]$, with the sampling interval Δ, where T = {t_i_}_i=1,2,…_, is the start time for each sampling window and $t_{i+1}-t_{i}\geq L$. The signal digitization with the discrete sampling window method is shown in Figure 3.

Time and frequency-based analysis methods are also used with the time series sensor data collected during the manufacturing process for condition monitoring. The signal analysis for failure detection does not only focus on all global properties of a signal in scope but also on information about local features of the signal, such as changes in frequency. Since changes in environmental and operating conditions can influence the vibration sources of the anomaly detection and make the signals non-stationary with a changing frequency and amplitude characteristics, traditional Fourier spectral analysis, such as fast Fourier transform (FFT) and short-time Fourier transform (STFT), cannot adequately be applied to non-stationary signals [18,19]. In essence, Fourier analysis is a global transform in both the time domain and frequency domain; therefore, it is unable to reveal the time-frequency local properties, which are the main features in the transient and non-stationary anomaly signals. However, the wavelet transform performs multiresolution analysis to extract the local features of the signals over time with the adaptive time-frequency analysis.

**Figure 4:**
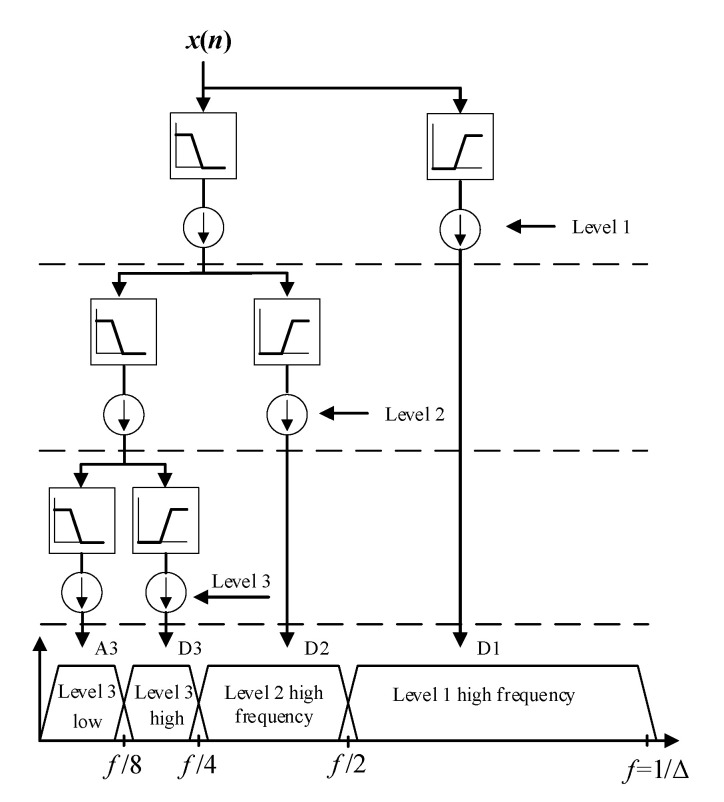
Discrete wavelets transform with 3 levels of high-pass and low-pass filter banks.

In our study, we apply the discrete wavelet transform (DWT) [20] to decompose the collected sensor signal in each sampling window $S\left(t_{i}\right)$. The discrete wavelet transform decomposes the given signal into a number of sets, where each set is a time series of coefficients at each decomposed level describing the time evolution of the signal in the corresponding frequency band. The signals are represented through a combined series expansion, namely, the approximation and the detail coefficients by the transform. We investigate the transform as the processing method used for the extraction of the features from the frequency information and location information. Figure 4 shows the discrete wavelet transform with three levels of high-pass and low-pass filter banks.

Relative wavelet energy describes information about the relative energy associated with different frequency ranges and can be considered a time-scale density [21,22]. This method can detect the similarity between the phase periods of the signals. The relative wavelet energy $R^{j}$ at the decomposed level *j* is defined as follows:

(1) $$R^{j}=\frac{E_{j}}{E_{total}}$$

with the energy of the signal at each decomposed level *E_j_* described as follows:

(2) $$E_{j}=\underset{k}{\sum }{\left|C_{j}\left(k\right)\right|}^{2}$$

The total energy for all levels is defined by the following:

(3) $$E_{total}=\underset{j}{\sum }\underset{k}{\sum }{\left|C_{j}\left(k\right)\right|}^{2}$$

where *C_j_*(*k*) is the DWT coefficient at the decomposed level *j*. The relative wavelet energy is used as the input feature to the prognostic algorithm. The calculated relative wavelet energy features are passed from the edge layer for anomaly detection on the cloud.

### 3.2. Proposed Prognostic Method Based on LSTM Model

Long short-term memory (LSTM) is a type of deep neural network algorithm [23,24], with the capability of feature learning based on recurrent neural network (RNN) architecture. In addition, the LSTM is a probabilistic recurrent neural network, which includes an input gate, a forget gate, a control gate, and an output gate; it is well-suited to learning the long-time lagged features from the time series. We used the LSTM model for anomaly detection in the time series in our proposed condition monitoring system, given its strong time series data processing ability.

A sequence of the relative wavelet energy R(t_i_) is used as the input of the LSTM, and each input data point is an *m*-dimensional vector of the relative wavelet energy at the time instance t_i_, which is generated from the edge layer. The LSTM model predicts the output vector ${\tilde{R}}_{T}$ with the extracted temporal evolutionary information from the input sequence. The temporal evolutionary information contains information about the machine condition with the aging process so that the anomalies can be extrapolated.

In our proposed LSTM network, we concatenate multiple LSTM units and a final fully connected layer with a stacked multilayered structure. Each LSTM layer has three inputs, including the previously hidden state $h_{T-1}$, cell state $c_{T-1}$, and current input $R_{T}$, and the hyperbolic tangent (tanh) activation function is adopted to process the output to be passed to the subsequent layer. The last fully connected layer is formed by *m* hidden neurons with a softmax activation function to produce the predicted output ${\tilde{R}}_{T+1}$ to approximate $R_{T+1}$ as the prediction of the relative wavelet energy at time T + 1. In the training process, the mean squared error (MSE) is used as the statistical metric to evaluate the model performance.

### 3.3. Anomaly Detection with Euclidean Distance

At time t, the relative wave energy is calculated from the time series input, and the output of the LSTM model is the prediction of the relative wavelet energy ${\tilde{R}}^{j}\left(t+1\right)$ at time t + 1. The difference between the estimation and real value at time t + 1 is the prediction error.

The prediction errors of the signal relative wavelet energy are used as the measurement indicator of anomaly detection. The errors between predicted and real relative wavelet energy vectors can estimate the development trend of the machine condition by the analysis of the transient features of non-stationary signals, given that the wavelet energy changes reflect the development trend of the machine condition. The error can be defined as follows:

(4) $$E={\left\{R^{j}-{\tilde{R}}^{j}\right\}}_{j=1,\;2,\;\ldots ,M}$$

where $R^{j}$ is the relative wavelet energy at scale *j*.

**Figure 5:**
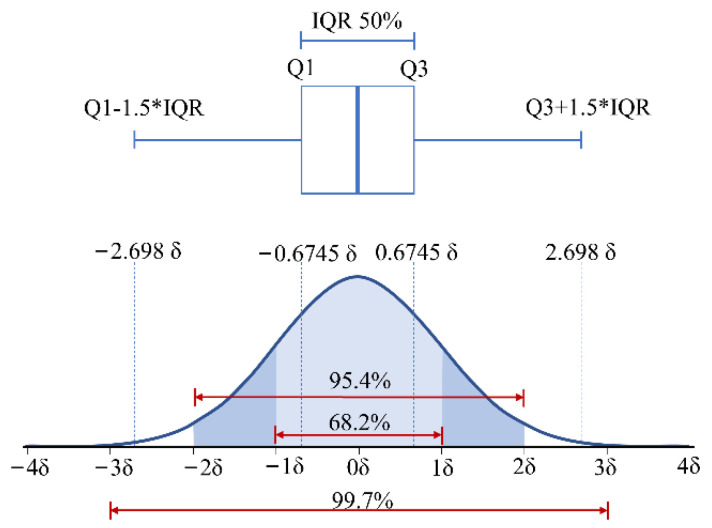
Interquartile range and empirical rule of a normal distribution.

With the statistical tests, the prediction errors can be modeled as a normal distribution with the standard deviation, δ. The prediction error of the anomaly point resides far away from the normal data points, which are the majority of all the data points. The interquartile range (IQR) and standard deviation are metrics to measure the spread of values in a dataset, as shown in Figure 5. The IQR does not consider all the data points in the dataset, and it is concerned more about the positions of the data. However, the standard deviation considers all the data points of a dataset, also including the outliers. In general, if the data point is not within three standard deviations of the mean value, it should be considered an outlier. According to the empirical rule, 99.7% of the data observed, following a normal distribution, lie within three standard deviations of the mean [25]. Therefore, the data points that are not within three standard deviations of the mean value are identified as anomaly data. 3δ will be used as the threshold for anomaly detection in our research.

## 4. Experiments and Results

### 4.1. Experiments Setup

**Figure 6:**
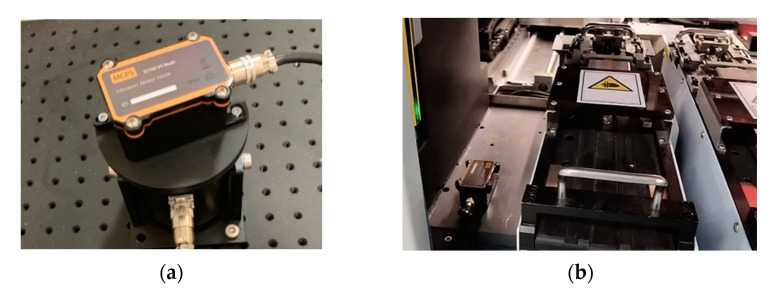
(**a**) Sensor node; (**b**) the IIoT sensor node installed in the component insertion machine.

This section verifies our proposed method by detecting machine anomalies with an edge-to-cloud condition monitoring system in a real workplace environment. The experimental work of the manufacturing machine condition monitoring has been conducted with an electronic component insertion machine, which is used for electronic product assembly by inserting the axial or radial electronic components and the functional parts of various shapes, as shown in Figure 6. Under the normal operation of the machine, we measured the acceleration time responses of the whole system via the vibration sensor nodes with MEMS accelerometers. The sensor nodes are wire connected to the IIoT gateway devices, with a sampling frequency of 1000 Hz. Figure 6a shows the sensor node used in the condition monitoring system, and the IIoT sensor node installed in the component insertion machine is presented in Figure 6b.

Electronic component insertion machines are used in electronics assembly by inserting various discrete components into printed circuit boards. The system needs a higher degree of stability for the system operating accuracy to improve manufacturing efficiency. However, environmental factors, such as larger ground and building floor vibrations or the misconduct of the operators and changes in the operating conditions, may cause equipment anomalies in the machine’s accuracy and affect the stability of the system. The anomaly detection of the vibration conditions in the operation of the machines is helpful in adjusting the parameters and calibrating the system in time.

According to the research on the generic vibration criteria used for vibration-sensitive technical facilities [26,27], such as electronic assembly and manufacturing facilities, the typical vibration frequency range is less than 100 Hz. Therefore, the proposed experiment system focused on the low-frequency vibration conditions.

### 4.2. Experiments Results

**Figure 7:**
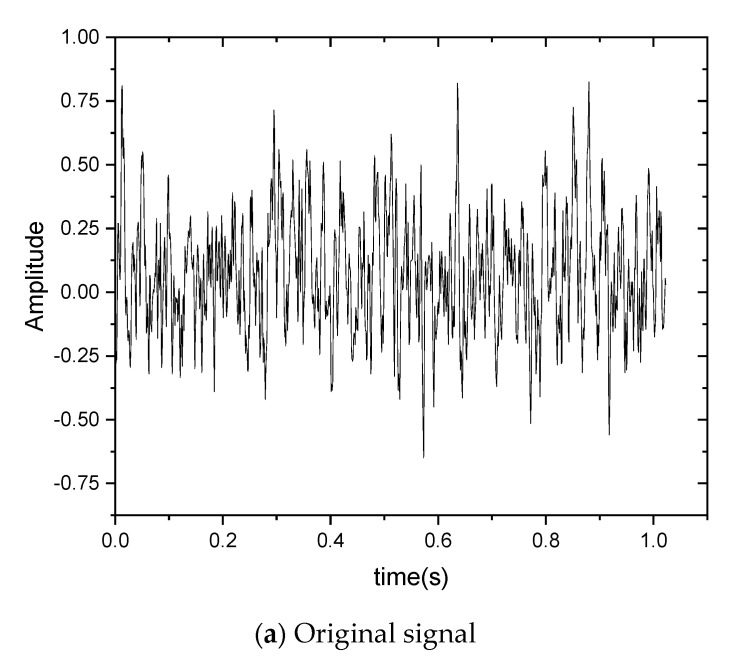

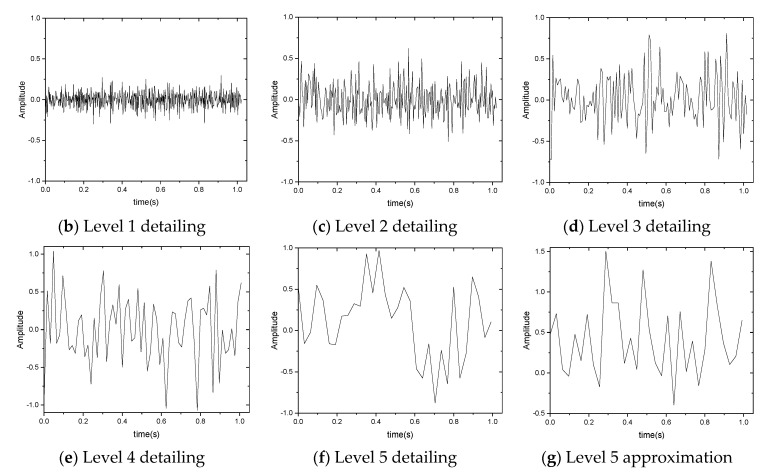
(**a**) Sensor vibration signal in one discrete sampling window. (**b**–**g**) Coefficients of the five-level approximation and detailing in wavelet decomposition.

Prior to calculating the relative wavelet energy to obtain the local features of the system’s running condition, the wavelet transform should be applied. Five wavelet transform decomposition levels were selected to achieve a good frequency resolution depending on how the system energy is distributed in the frequency domain and on the system’s complexity. Figure 7 shows the decomposition of the sensor vibration signal in one discrete sampling window from the IIoT sensor node in Figure 6b.

As shown in Figure 7, the discrete wavelet transform decomposes the input vibration sensor signal into five levels. The five detail coefficients are extracted from the decomposition levels with one approximation coefficient. The detail coefficients correspond to the high-frequency parts of each level. In addition, the approximation coefficient corresponds to the low-frequency part of the fifth level.

**Figure 8:**
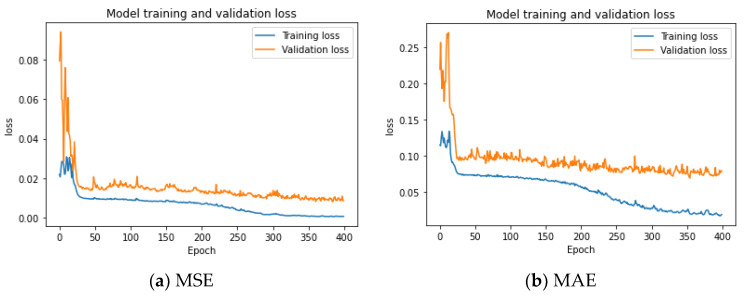
Training and validation loss function graph of the RWE–LSTM modeling process with (**a**) the Mean Squared Error loss function and (**b**) Mean Absolute Error loss function.

The relative wavelet energy for each level is calculated by Equation (1), then inputted to the LSTM to establish the optimal model for anomaly prediction in the condition monitoring system. The RWE–LSTM models are trained by an unsupervised method with training and validation datasets. The proposed model is a stacked LSTM with three subsequent layers and one fully connected layer. The function activation is the sigmoid function, and the model is set for 400 epochs. Considering the computational complexity and the model training performance, the Mean Squared Error (MSE) and Mean Absolute Error (MAE) loss functions are used for the model training and validation. The training and validation loss function graph for the LSTM model in the training period is shown in Figure 8. The training and validation loss curves decrease and become stable after 300 epochs. The performance and convergence behavior of the model shows that the MSE loss function is a better match for this model training.

**Figure 9:**
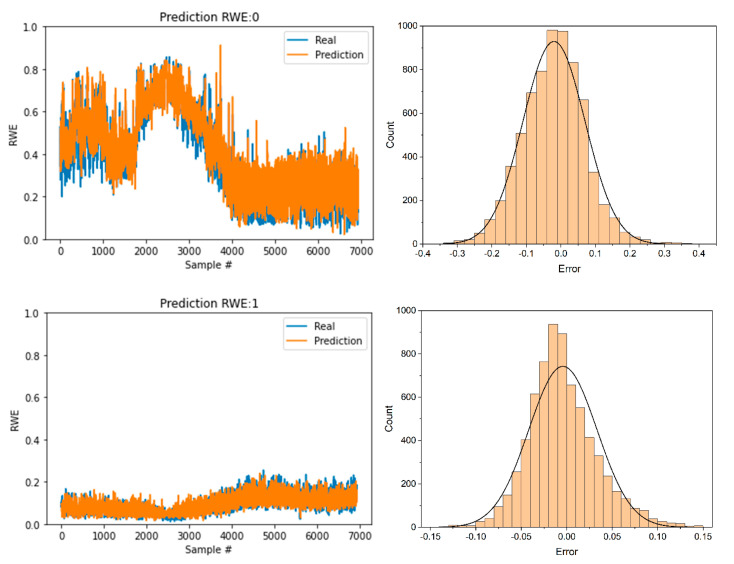

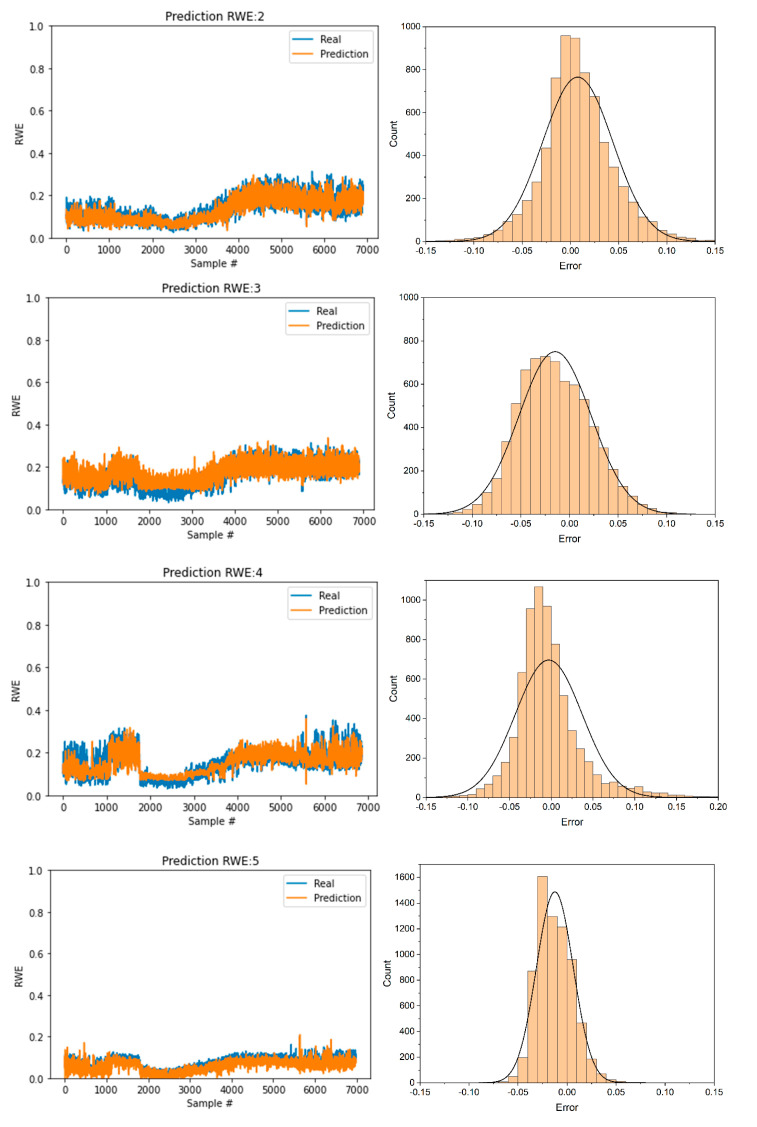
Results of the RWE–LSTM model prediction with five levels of the RWE (**left**) and histograms of the prediction errors with normal distribution analysis (**right**).

Figure 9 shows the prediction results for five levels of the RWE and error analysis of the proposed method on a sample time-series dataset. In the left column of Figure 9, the real-time-series data are shown in blue, whereas the predictions are shown in orange. In this plot, the proposed model is able to learn the time-series trend. In the right column of Figure 9, the histograms of the prediction errors are provided. The prediction error is modeled as a normal distribution. Table 1 presents the means and standard deviations of the normal distributions of five levels of the RWE prediction errors. According to the empirical rule, the anomaly detection threshold is defined as three times the standard deviation.

**Figure 10:**
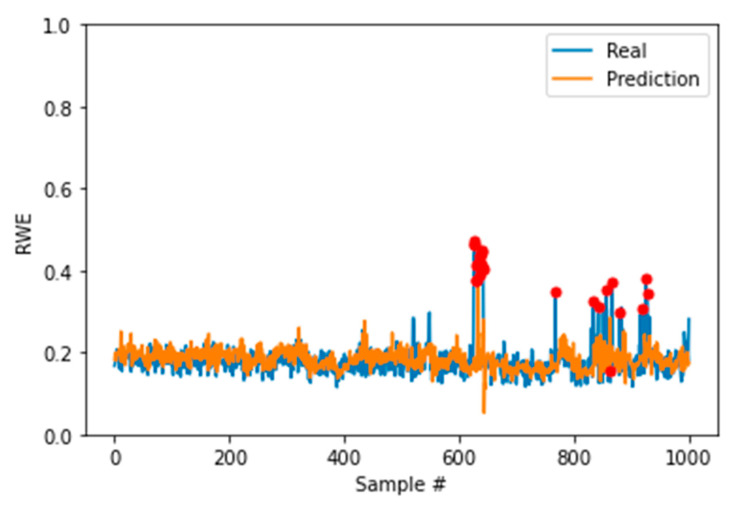
Anomaly detection results of the RWE–LSTM model (red dots mark the anomalies).

Figure 10 shows the anomaly detection, marked as the red dots in the plot, for the manufacturing machine condition monitoring data in the real running time with three times the standard deviation as the threshold. This finding demonstrates the effectiveness of the trained RWE–LSTM model.

## 5. Discussions

The current proposed IIoT architecture for condition monitoring is based on the edge-to-cloud scenario to reduce the data transmission and algorithm computing on the cloud servers efficiently. However, the IoT network’s architecture will face challenges since the number of sensors and the complexity of the application scenarios increase. There are various advanced network architectures proposed for the next generation of IoT applications, such as the Multiple IoT scenario (MIoT) [28,29] and Multi-access edge computing (MEC) [30]. So, the effective integration of edge devices with the new IoT architectures requires further investigation to implement more robust and low-latency applications in the industrial field.

## 6. Conclusions

In this paper, we presented an IIoT-based condition monitoring system with an unsupervised learning approach to detect anomaly conditions for manufacturing applications. The discrete sampling window method is applied in the IIoT gateway on the edge layer to reduce the network traffic overhead for the IIoT while maintaining detection accuracy. The unsupervised learning with the deep LSTM network module is used for the time series of the long-term condition monitoring in the manufacturing process. We demonstrated the proposed system’s architecture and algorithms on real manufacturing machines and showed that our condition monitoring hardware platform works stably and efficiently, and the anomaly detection model can detect unknown spatial and temporal anomalies on the manufacturing machines.

## Figures and Tables

**Table 1:** Normal distributions for five levels of the RWE prediction errors.

Components/Level	*A*5	*D*1	*D*2	*D*3	*D*4	*D*5
Mean	−0.02013	−0.00138	0.00754	−0.01489	−0.00303	−0.01243
δ	0.08989	0.0344	0.03638	0.03713	0.03999	0.01871
3δ	0.26967	0.1032	0.10914	0.11139	0.11997	0.05613

## Data Availability

The data presented in this study are available on request from the corresponding author. The data are not publicly available due to system security issues.
